# Muscle composition and the imminent mortality risk after hip fracture

**DOI:** 10.1002/jcsm.13090

**Published:** 2022-10-19

**Authors:** Ling Wang, Lu Yin, Minghui Yang, Xiaoguang Cheng

**Affiliations:** ^1^ Department of Radiology Beijing Jishuitan Hospital Beijing China; ^2^ Medical Research & Biometrics Center National Center for Cardiovascular Disease Beijing China; ^3^ Departments of Traumatic Orthopedics Beijing Jishuitan Hospital Beijing China

Skeletal muscle density, measured by computed tomography (CT) as the mean attenuation in Hounsfield Units (HU) (also termed myosteatosis), is well established as a biomarker for predicting mortality and treatment‐related outcomes in cancers,^1^ heart failure,^2^ frailty fractures and other diseases.^3^, ^4^, ^5^ We therefore read with interest the paper ‘Subcutaneous fat area at the upper thigh level is a useful prognostic marker in the elderly with femur fracture’ by Kim *et al*.^6^ The authors presented an interesting study on the impact of body tissue composition at the upper thigh level on 1‐year mortality in elderly patients with a proximal femur fracture. The paper showed that subcutaneous fat area but not muscle size or density was positively associated with 1‐year mortality. The authors highlight the importance of the upper thigh subcutaneous fat area as an independent prognostic marker for elderly hip fracture patients that could be highly useful for planning personalized nutritional support and rehabilitation interventions to reduce mortality. However, the reported results regarding muscle parameters raise some questions worth addressing.

The authors failed to mention when the CT images were acquired, that is, within a short time after the injury or following surgery. The study results might be affected by changes in body composition during bed rest or surgery. Furthermore, this study measured both thighs, whereas the muscles on the fractured side would swell after the hip fracture, and the density and muscle area would change accordingly.

Another concern about the study is the scan protocol for the pelvic CT scans. The tube voltage ranged from 120 to 130 kVp, which would introduce variations in the muscle density assessment. As is well known, mean muscle tissue attenuation measured in HU increases significantly at lower kVp.^7^ Furthermore, lower tube currents introduce more noise on CT images and lead to increases in the measured muscle density.^8^ The impact of variations in CT acquisition parameters on muscle measurements needs to be considered in the interpretation of the results and further adjusted in the statistical analysis.

Figure 3 in the paper by Kim *et al*.^6^ raised a further concern about the interpretation of the study. The vastus medialis muscle of the right thigh in figure 3B appears much larger than that of the left thigh. This figure gives the impression that an elderly woman with greater muscle mass and lower fat content has a higher risk of mortality, which is contrary to the general health consensus. In addition, there are no reports about interquartile range of the muscle density in survivor and non‐survivor groups in table 2.

We have performed a prospective study in a Chinese cohort of 374 hip fracture patients who underwent hip CT scans immediately (<48 h) after injury during 2015–2016 and were followed up for a median of 4.5 years. Of these patients, 24 died within the first year of having a hip fracture. Cross‐sectional area (CSA) and density of the gluteus maximus (G.max) were measured at the level of the greater trochanter and of the gluteus medius and minimus (G.med/min) muscles at the level of the third sacral vertebra. Compared with the patients who died, most muscle parameters were statistically significantly higher in surviving patients, except for G.med/min muscle area. Muscle density of G.MaxM (adj. HR 1.80; 95% CI, 1.06–3.06) and G.Med/MinM (adj. HR 1.89; 95% CI, 1.11–3.24) predicted mortality in the first year after hip fracture (*Figure* 1), but other muscle variables did not.

**Figure 1:**
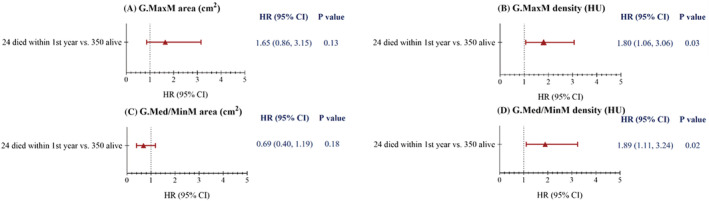
Adjusted* hazard ratios of death risk per one SD decrease of G.MaxM area (A), G. MaxM density (B), G.Med/MinM area (C) and G.Med/MinM density (D) with time frame. *Adjusted for age, sex and Parker score before hip fracture.

## Conflict of interest

None.

## Funding

This work is supported in part by the National Natural Science Foundation of China (Grant No. 81901718) and Beijing Hospitals Authority Clinical Medicine Development of Special Funding Support (code: ZYLX202107).
